# Supplementary material for: Evaluation of diversity indices to estimate clonal dominance in gene therapy studies
Source: Mol Ther Methods Clin Dev. 2023 May 9;29:418–25. doi: 10.1016/j.omtm.2023.05.003 (PMC10220254; doi:10.1016/j.omtm.2023.05.003)
Supplement: Document S1. Figures S1–S4 [file mmc1.pdf]

OMTM, Volume *29*

Supplemental information

Evaluation of diversity indices to estimate clonal dominance in gene therapy studies

Guillaume Corre and Anne Galy

Supplemental Materials

Supplemental Figures

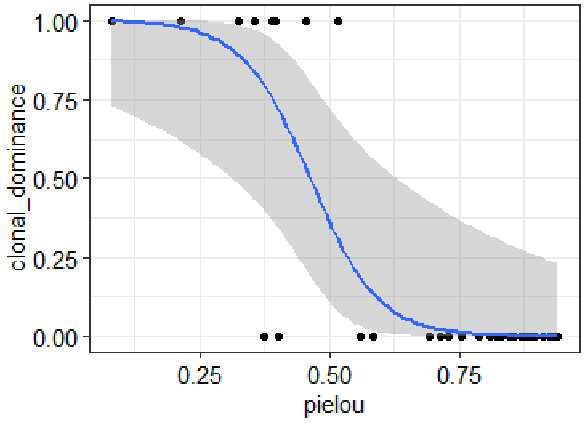

Figure S1 : Regression analysis of Pielou data in Figure 1. A logistic regression curve was generated from the values of the Pielou index from Figure 1 plotted against the clinical status (clonal dominance = 1 in case of leukemia or myelo-dysplastic syndrome, and clonal dominance = 0 ni case of no adverse event). The shaded grey area represents the 95% confidence interval of the regression.

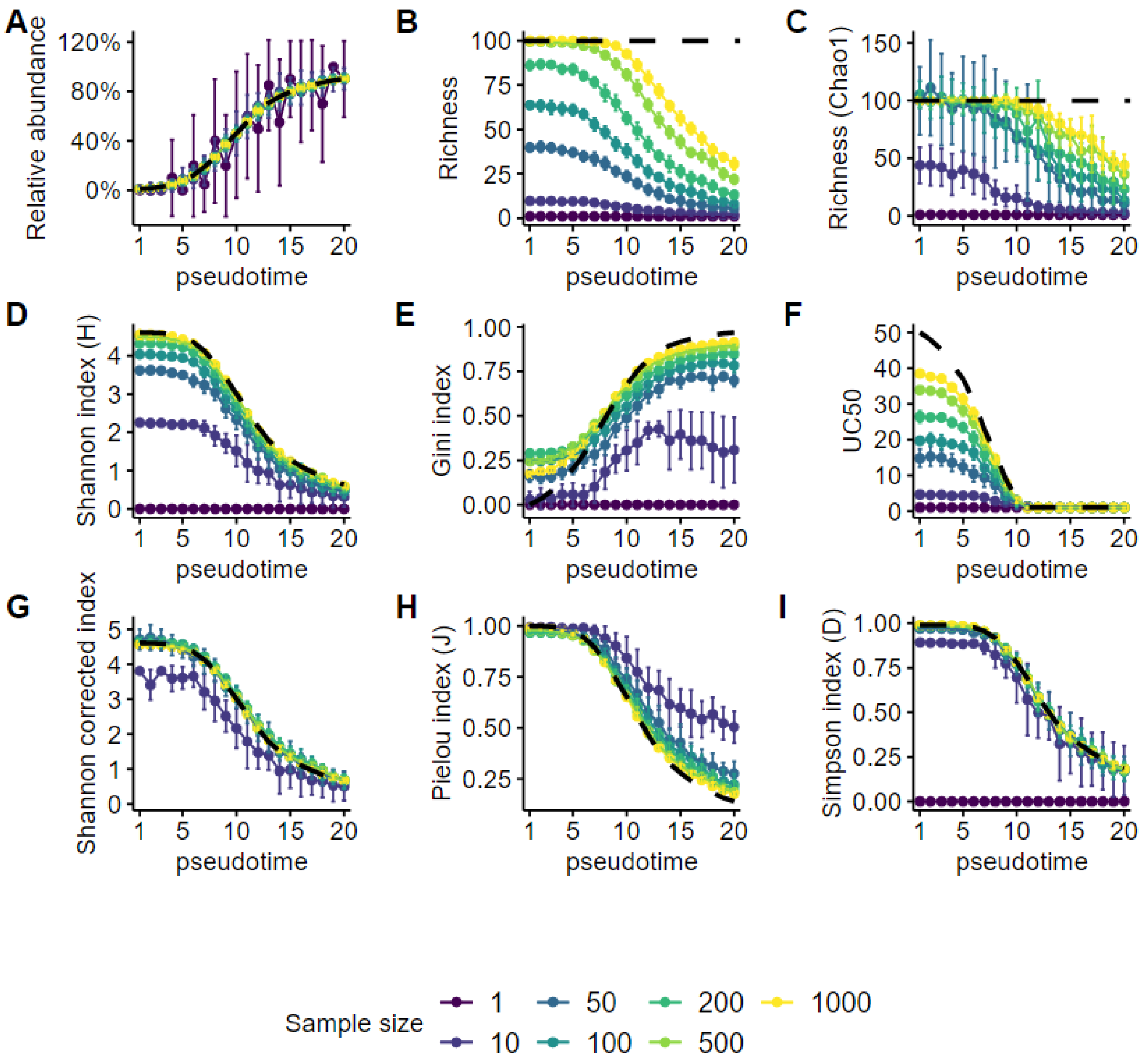

Figure S2: Simulation of a clonal dominance and evaluation of diversity indices with a sample richness of 100 clones. The different parameters are as in Figure 2.

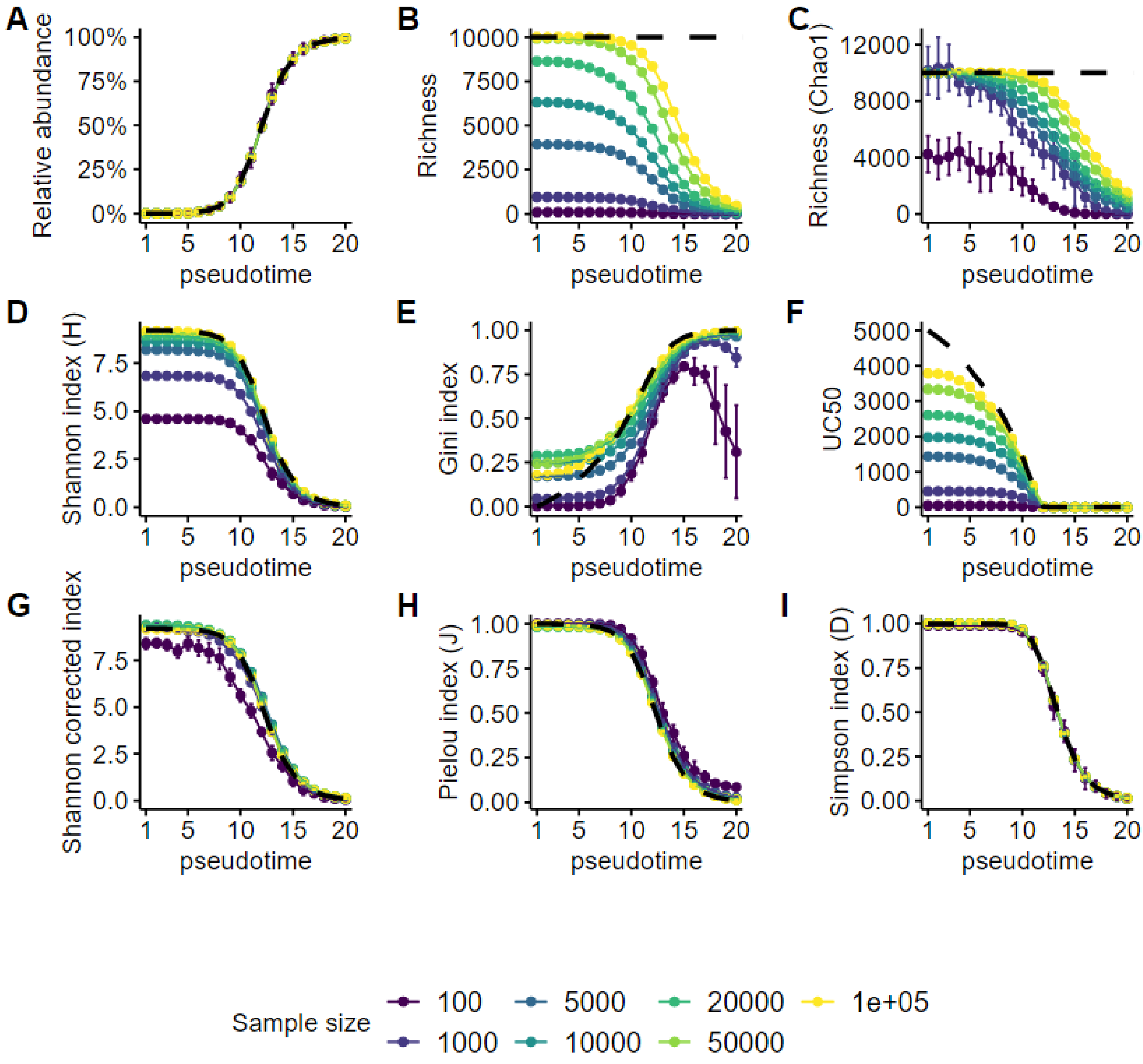

Figure S3: Simulation of a clonal dominance and evaluation of diversity indices with a sample richness of 10,000 clones. The different parameters are as in Figure 2.

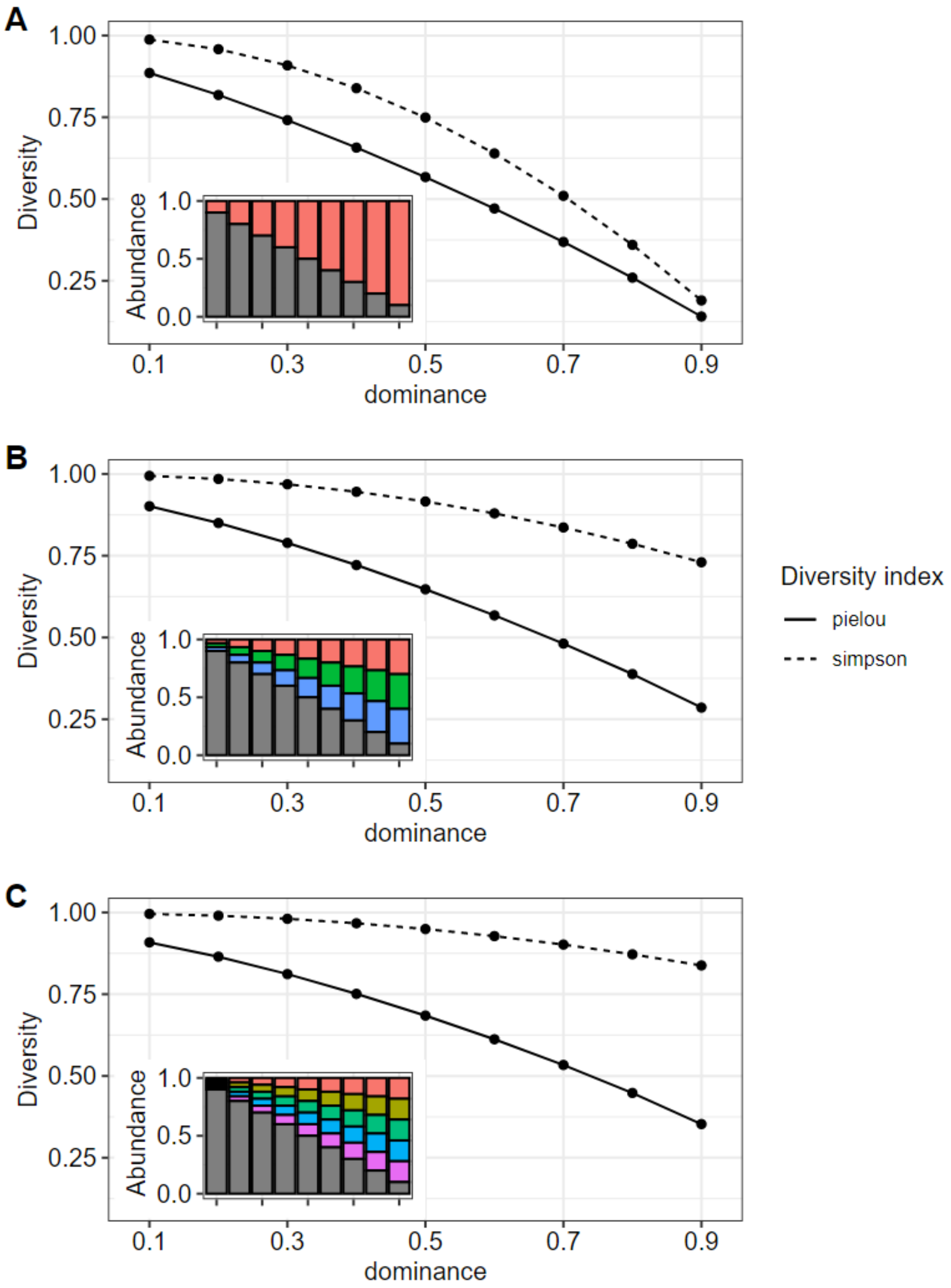

Figure S4 : Comparison of evenness using Pielou and Simpson indices in a population of IS with variable clonal abundance. The IS data set was described in [25]. Panels A-C present the behavior of Pielou and Simpson indices for different numbers of simulated dominant clones: 1 clone (panel A), 3 clones (panel B) and 5 clones (panel C). Inserts present the contribution of dominant clones in color and of the other clones are in grey.
