# Supplementary material for: Evaluation of diversity indices to estimate clonal dominance in gene therapy studies
Source: Mol Ther Methods Clin Dev. 2023 May 9;29:418–25. doi: 10.1016/j.omtm.2023.05.003 (PMC10220254; doi:10.1016/j.omtm.2023.05.003)
Supplement: Document S2. Article plus supplemental information [file mmc2.pdf]

Molecular Therapy
Methods & Clinical Development
Original Article

Evaluation of diversity indices to estimate clonal dominance in gene therapy studies

Guillaume Corre[1,2] and Anne Galy[1,2]

[1]Genethon, 91000 Evry, France; [2]Université Paris-Saclay, University Evry, Inserm, Genethon, Integrare Research Unit UMR_S951, 91000 Evry, France

In cell and gene therapy, achieving the stable engraftment of an abundant and highly polyclonal population of gene-corrected cells is one of the key factors to ensure the successful and safe treatment of patients. Because integrative vectors have been associated with possible risks of insertional mutagenesis leading to clonal dominance, monitoring the relative abundance of individual vector insertion sites in patients' blood cells has become an important safety assessment, particularly in hematopoietic stem cell-based therapies. Clinical studies often express clonal diversity using various metrics. One of the most commonly used is the Shannon index of entropy. However, this index aggregates two distinct aspects of diversity, the number of unique species and their relative abundance. This property hampers the comparison of samples with different richness. This prompted us to reanalyze published datasets and to model the properties of various indices as applied to the evaluation of clonal diversity in gene therapy. A normalized version of the Shannon index, such as Pielou's index, or Simpson's probability index is robust and useful to compare sample evenness between patients and trials. Clinically meaningful standard values for clonal diversity are herein proposed to facilitate the use of vector insertion site analyses in genomic medicine practice.

INTRODUCTION

Gene transfer vectors derived from lentiviruses or retroviruses have been successfully used for the cell and gene therapy of rare genetic disorders including primary immune deficiencies, hemoglobin disorders, cytopenias, or neurodegenerative diseases[1] and for cancer immunotherapy with T cell receptor or chimeric antigen receptor (CAR)-T cells.[2,3] As vectors stably integrate into the genome of target cells, their genomic insertion sites (ISs) provide important biological and safety information on the effects of gene therapy. ISs inform on the genomic and epigenetic positional preferences of vectors[4] and enable cellular lineage ontology studies,[5] estimations of number of engrafted transduced cells,[6] and *in vivo* hematopoiesis reconstitution in patients[7] or in animal models.[8] The diversity of genomic ISs describes the polyclonal nature of the gene-modified cell population, which is an important safety assessment considering the clinical risks of insertional mutagenesis caused by retroviral vectors.[1,9] In hematopoietic stem cells (HSCs), the insertions of retroviral vectors using active long terminal repeat (LTR) promoters have caused the transactivation of proto-oncogenes resulting in clonal dominance and leukemic events.[10] Self-inactivated lentiviral vectors (LVs) have reduced this risk, but LVs can still disrupt gene expression in HSCs or in T cells, resulting in proliferative advantages and clonal dominance without transformation, as observed in some patients treated with LVs for thalassemia[11] or with CAR-T cells for cancer.[12,13] Consequently, vector genomic IS analyses have become integrated into patient safety monitoring protocols when using integrative gene therapy vectors.

Yet, the measure of gene therapy vector ISs is complex and not a standard assay in medical biology or genomic medicine practice. Methods for IS sequence retrieval and for DNA sequencing have largely evolved over the last decades to increase coverage, output, and speed.[14] Bioinformatic analyses yield multiple results that include genomic mapping data, information relative to ISs near specific sets of genes like oncogenes, estimation of cellular lineage relationships, measures of gene-modified cell counts, and measures of population diversity. Statistical methods for measuring clonal diversity mostly originate from the ecology field[15] and consider that cells sharing the same unique IS (also called clones) are species of which we want to measure the number and the relative abundance. Richness (the number of different clones) can estimate gene therapy efficacy, whereas the absence of any dominant species of clone over time (high evenness) can define gene therapy safety. Many indices are used to estimate clonal diversity either by the relative abundance of gene-modified cells with Gini or Shannon indices or by estimating population size with the Chao1 index or the number of clones in the top 50% of cell abundance with the UC_{50} value, often reported together in gene therapy studies.[16–23]

The Shannon index of diversity (or entropy) is a popular diversity index for gene therapy studies. It considers both the richness and the evenness of the sample but does not allow one to compare samples with different richness. It is sensitive to the amount of DNA, the number of vector copies in the sample, and the sequencing depth.[24] It also assumes that all species are represented in a sample and are randomly sampled. Moreover, the formula contains logarithms, but different bases are used in the literature (i.e., base 2[23]), so inter-study

Received 1 September 2022; accepted 4 May 2023;
https://doi.org/10.1016/j.omtm.2023.05.003.

Correspondence: Anne Galy, ART-TG, Inserm US35, 30 rue H. Desbruères, Corbeil-Essonnes 91100, France.
E-mail: anne.galy@inserm.fr

 © 2023 The Authors.
This is an open access article under the CC BY license (http://creativecommons.org/licenses/by/4.0/).

Table 1. Description of the gene therapy trials analyzed in the present study

Study	Aiuti et al., 2013[17]	BIFFI et al., 2013[18]	Braun et al., 2014[19]	Magnani et al., 2022[22]	Wang et al., 2010
Disease	WAS	MLD	WAS	WAS	SCIDX1
Number of patients	3	3	10	8	8
Vector	SIN-LV	SIN-LV	MLV derived	SIN-LV	MLV derived
Pseudotype	VSVG	VSVG	GALV	VSVG	Ampho
Transgene promoter	w1.6 WASp	PGK	Full LTR	w1.6 WASp	Full LTR
Transgene	WAS	ARSA	WAS	WAS	IL2RG
PCR method for IS library	LAM-PCR	LAM-PCR	nrLAM-PCR and LAM-PCR	LM-PCR	LM-PCR
Sequencing technology	454 and MiSeq	454 and MiSeq	454 and MiSeq	MiSeq	454

WAS, Wiskott Aldrich syndrome; MLD, metachromatic leukodystrophy; SCID X1, X-linked severe combined immune deficiency; SIN-LV, self-inactivated lentiviral vector; MLV, murine leukemia virus; LTR, long terminal repeat; LAM-PCR, linear amplification mediated PCR; nr-LAM-OCR, non-restrictive variant LAM-PCR; LM-PCR, ligation-mediated PCR; 454, 454 Roche DNA pyrosequencing; MiSeq, Illumina MiSeq DNA sequencing.

comparisons of the Shannon index diversity in gene therapy must be made with caution, especially in samples of different sizes. The use of a normalized index would be preferable, enabling comparisons in studies with different size datasets. A normalized version of the Shannon index, also known as Pielou's index,[25] is used to estimate evenness. Simpson's index is another measure of diversity taking into account the number of species present, as well as the relative abundance of each species.

We herein compared the use and performance of these different indices that estimate relative abundance for the analysis of IS diversity in gene therapy studies in which some adverse events occurred or not. Gene therapy trials for the treatment of Wiskott Aldrich syndrome (WAS), metachromatic leukodystrophy (MLD), or X-linked severe combined immunodeficiency (SCID-X1) have used the Shannon index to evaluate the polyclonality of the gene-corrected cell populations in patients over time.[17–20,22,23,26] We modeled the performance of these indices upon the occurrence of clonal dominance and found differential impacts of sample size on the results obtained. Using an already published experimental IS dataset with spiked clones,[27] we show that Pielou's index provides a sensitive measure of clonal dominance. Using published gene therapy studies, we also show that the Pielou index enables inter-study comparisons and can be used to define a safety threshold in trials where leukemias occurred. Finally, we used Pielou's and Simpson's indices to demonstrate the sustained clonal diversity in a gene therapy clinical trial for WAS.[6,20,22,26]

RESULTS

Published datasets demonstrate the interest of using a normalized diversity index

The relative abundance of gene-modified cells was analyzed in several published gene therapy clinical trial datasets (Table 1). Two of these trials used gamma-retrovirus-derived vectors, which have induced proliferative disorders in some patients.[19,23] In other trials, an SIN-lentiviral vector was used to treat WAS and MLD patients,[17,18,20,22,26] and no clonal dominance was observed. Figure 1 presents the longitudinal evolution of several indicators in these published studies. For clarity, we only presented the diversity analysis of peripheral blood mononuclear cells (PBMCs) or myeloid cell samples. The number of unique ISs varies over a wide range of values and among studies, patients, and time points. It is therefore obvious that the unique IS value is insufficient as a single indicator to estimate the polyclonality of gene-modified cells (Figure 1A). The Shannon index also varies between studies (Figure 1B). The Shannon index value drops in patients when a clonal dominance occurs (between 20% and 98% of cells are leukemic blasts at the indicated time points circled in red in Figure 1B).[19,23] In the Wang et al. study, diversity was restored upon chemotherapy treatment in patients 7 and 10, and the Shannon index value rose. However, Shannon index values fluctuated in other patients and other studies without evidence for clonal dominance. The values of this indicator were also found to be very variable not only between patients but also during the follow-up of each patient, ranging from 3.4 to 8.8 in the Magnani et al. study in which no clonal dominance occurred.[22] Patient-to-patient fluctuations in this index are mostly related to the richness of samples, as shown in Figure 1A, which may be the consequence of different sampling size, differences in the methodology used to identify ISs, or the efficacy of cell transduction. In the Wang et al. and Braun et al. studies,[19,28] fewer than a thousand clones were identified per sample because DNA pyrosequencing technology was used. Instead, Illumina next-generation DNA sequencing used in the other studies (Table 1) generates up to 10,000 unique ISs per sample and therefore larger values of the Shannon index. Figure 1C shows the advantage of using Pielou's normalized Shannon index, which was calculated here from values of Shannon and ISs to control for differences in sample richness. Pielou's index shows that evenness is stable in healthy patients over time, confirming that changes in the Shannon index were related to different richness. For patients with adverse events, the evenness drops rapidly as expected and discriminates more obviously the healthy and leukemic patients. The control of the sample evenness using this normalized index makes it possible to define a threshold value below which it would be a health concern for patient. According to the values observed in patients with leukemia and healthy patients across the different trials, a threshold value of Pielou's index at 0.5

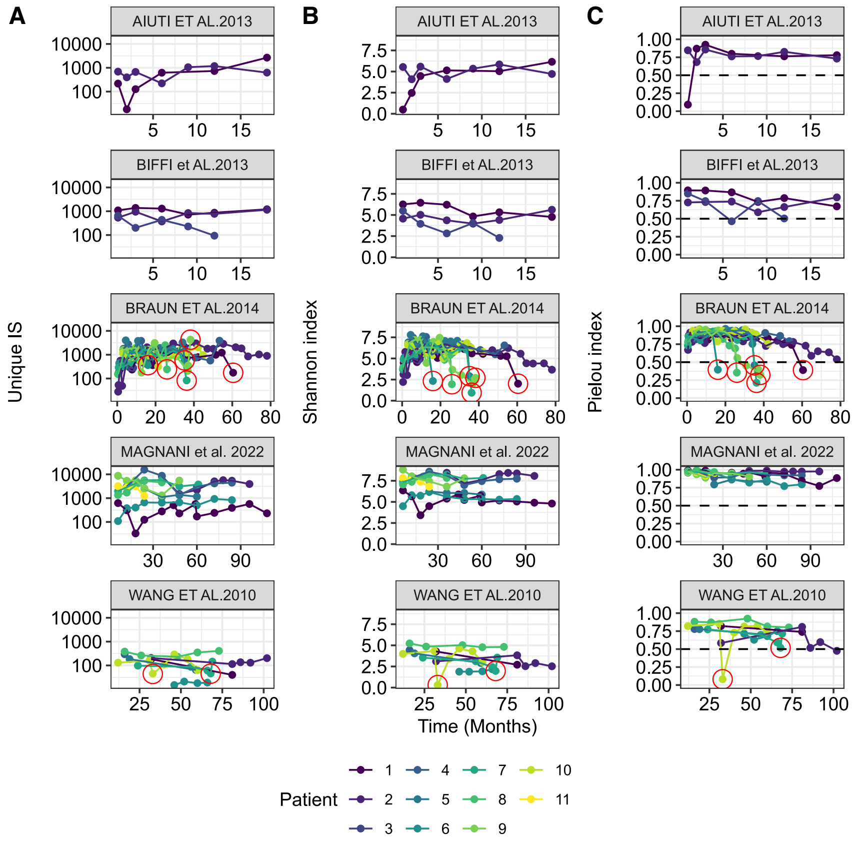

Figure 1. Patient samples IS longitudinal analyses according to different indicators

(A) shows the number of unique ISs, (B) shows the Shannon index of diversity (log base e), and (C) shows the calculated Pielou's index (normalized Shannon index) for different published *ex vivo* gene therapy trials as indicated on top of graphs (alphabetically) and as listed in Table 1. In some graphs, red circles indicate the reported diagnosis of adverse events such as lymphoproliferative disorders. Patients' identification numbers were attributed incrementally and iteratively for each study so that all panels share the same legend for patient identification.

seems to adequately define low- and high-diversity samples (dashed horizontal line in Figure 1C). Below this threshold, there is an increasingly high probably of clinically relevant clonal dominance as shown in a logistic regression analysis (Figure S1). This does not take into account asymptomatic patients. One of the patients in Braun et al.[19] and one patient in Wang et al.[23] show that evenness is decreasing over time reaching the threshold. These two patients have high frequency of clones with an IS in MDS1 and CCND2 genes, respectively, but no adverse event diagnosed at the last reported time point.

Computer simulation of clonal dominance and evaluation of the performance of diversity indices

In order to evaluate the effectiveness of several richness and evenness indices, we simulated a polyclonal population with an increasing clonal dominance over time (Figure 2). A polyclonal population of 1,000 clones (richness indicated by a dashed line in Figure 2) was simulated using a random proliferation rate for each clone (see simulation details in materials and methods). Supplemental Figures S2 and S3 show the same simulations for 100 or 10,000 clones, respectively.

Independently of the true population richness and sample size, we observed that the estimated proportion of the "dominant clone" in the different samples is a good estimation of its abundance in the population (Figures 2A, S2A, and S3A) even if small samples tend to be more variable when abundance is around 50%, as expected. Similarly, when sample size is large enough compared with population richness, all the clones are detected at least once in each sample for early time points (Figures 2B, S2B, and S3B). Increasing sample size increases the number of unique detected clones up to saturation (e.g., all possible clones detected). The simulation shows that above 20% of clonal dominance in the population, the estimated richness is negatively biased even for large samples (Figures 2B, S2B, and S3B).

Different indices have been proposed to estimate population richness even from small samples. One of them is the Chao1 index that takes advantage of the presence of rare species to estimate unseen species.[29] Figures 2C, S2C, and S3C show that the Chao1 index can correctly estimate the population richness even for small samples but only when the evenness is relatively high (early pseudotime points) and up to a moderate 15% clonal dominance if sample size is large.

The frequently used Shannon index decreases with the appearance of clonal dominance (Figures 2D, S2D, and S3D). However, because the Shannon index considers both the richness (which increases with sample size) and the evenness in the calculation, we also observed that its value increases with sample size (compare Figures 2D, S2D, and S3D for results in different sample size). Comparing different values of diversity with the Shannon index is then risky if sample size/richness is not equivalent between samples. Two other indices have been proposed in gene therapy monitoring of cell polyclonality.

The Gini index was developed in the field of economy to evaluate the amount of inequality in the contribution of different entities. Here, cell clones that have the same abundance contribute equally, and the Gini index is close to 0. If one clone becomes dominant, it will skew the frequency distribution, and the Gini index becomes close to 1. Figures 2E, S2E, and S3E show that the Gini index is influenced by sample size (relative to population richness) and is biased even at early pseudotime points.

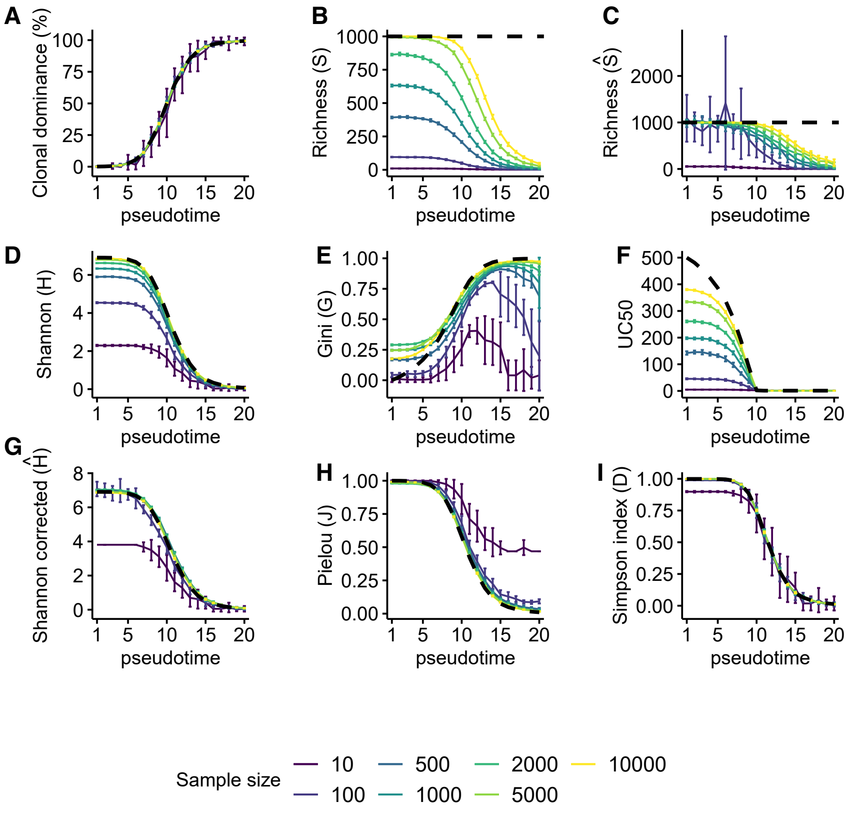

Figure 2. Simulation of a clonal dominance and effects of sample size on diversity indices

A computer model was generated to simulate random sampling in a population of 1,000 clones that are equally abundant at T0. Each clone has a random proliferation rate, and one "dominant" clone is given a proliferation rate four times larger than the others. Random samplings of this population are performed with 50 replicates per sampling size and per time point. Results include standard deviation error bars, and the population value is represented as a dashed black line. Results represent (A) the relative abundance (proportion) of the dominant clone in the population, (B) sample richness, (C) Chao1 richness estimation, (D) Shannon index, (E) Gini index, (F) UC_{50} index, (G) bias-corrected Shannon index using coverage and Horvitz-Thompson correction, (H) Pielou index, and (I) Simpson index. Different sampling sizes ranging from 10 to 10,000 are represented by the indicated color codes that are shared in all panels.

Another index proposed[12,16,30] is the UC_{50} value expressing how many clones contribute to the most expanded 50% of the total cell abundance. Figures 2F, S2F, and S3F show that UC_{50} rapidly drops to 1 when the dominant clone reached 50% of relative abundance and is sensitive to sample size (and richness estimation). Thus, comparing two samples is then difficult without considering the richness.

In order to remove sample size/richness effect, one strategy is to correct the Shannon index value using an estimation of the true sample richness. A proposed solution is to use a correction based on sample coverage and the Horvitz-Thompson method (Figures 2G, S2G, and S3G).[31] Sample size effect is removed except for very small samples, and the corrected value is still influenced by the population richness.

Another approach to remove sample size/richness effect is to normalize the Shannon index by the maximum value expected if the evenness was perfect between identified clones, which corresponds to Pielou's index in the literature.[25] Figures 2H, S2H, and S3H show that Pielou's index ranges from 1 for even clonal abundance to 0 when the clonal dominance has reached its maximum value. Pielou's index is very close to the population value (dark dashed line) even though the value is slightly overestimated at late pseudotime points and when sample size is very small. Pielou's index values are independent of the richness and may be used to compare different samples with different sampling efforts or different true richness. Depending on the species abundance distribution shape, Pielou's index is dynamic and takes intermediate values that drop only in the case of a significant clonal dominance (>50% in this simulation).

Simpson's dominance index is another estimator of sample evenness that gives more weight to abundant species. It expresses the probability that two independent sampled cells taken at random from a dataset belong to the same clone.[15] The less diverse the population is, the higher is this probability. Figures 2I, S2I, and S3I show that Simpson's index performs similarly to Pielou's but is less biased independently of sample size and richness.

The three most robust indices for our purpose, Shannon's, Pielou's, and Simpson's, were computed in simulations with increasing richness and clonal abundance. In the simulations, one clone is dominant, and other clones' abundance is distributed using a random uniform distribution. Surface plots in Figure 3 clearly show the dependence of the Shannon index on sample richness, whereas Pielou's and Simpson's indices are almost independent from the richness except when really small. Thus, both the Pielou and Simpson indices are almost linearly dependent on the amount of clonal dominance, with all others clones being distributed following a random uniform distribution.

Comparison of Pielou and Simpson indices in real conditions

In order to evaluate the behavior of the Simpson and Pielou indices in experimental conditions, we used a dataset of ISs with different amounts of clonal dominance from a previously reported study.[27] To artificially increase clonal dominance, a DNA from a polyclonal population of LV-transduced cells was spiked with known and increasing quantities of DNA extracted from stable cell clones containing either one, two, or three copies of LV, generating increasing amounts of multiple detectable ISs. Under these conditions, an

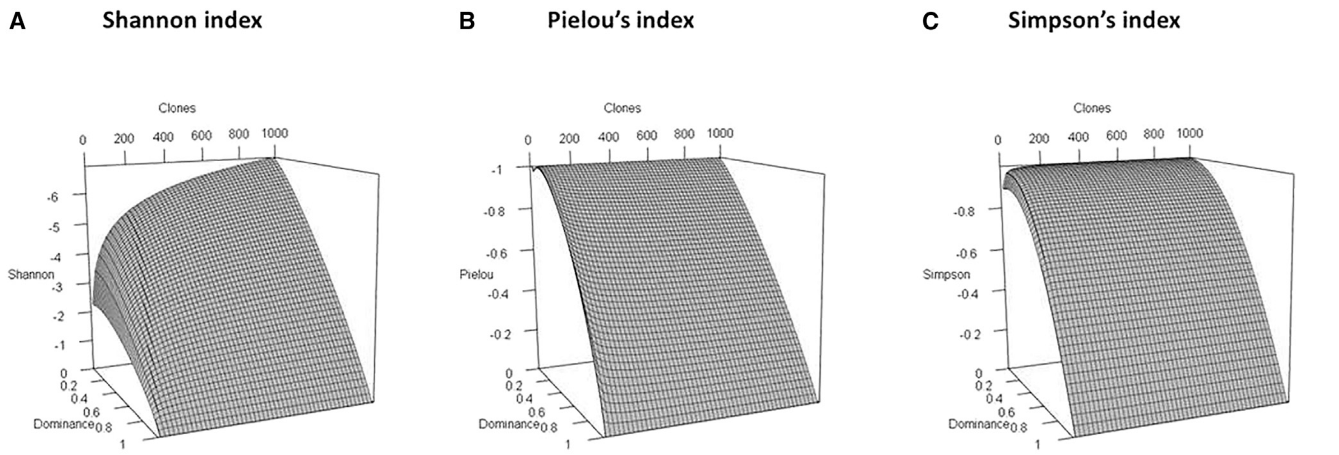

Figure 3. Evaluation by different indices of clonal dominance according to richness

Surface plots were generated by computer simulation using different richness (0–1,000) and dominance (0–1) values to represent their influence on the Shannon (A), Pielou (B), and Simpson (C) indices.

average of 900 ISs (683–920) for 2,500 estimated cells per condition (2,388–3,502) were analyzed. The contribution of major ISs is represented in Figure 4. Both Simpson's and Pielou's indices detected the drop in diversity due to the increased clonal dominance, but Pielou's index seemed to be the most sensitive to the change in evenness over the gradually increasing dominance (Figure 4). Figure S4 confirmed that the Simpson index is less sensitive to the increase in dominance than Pielou, especially when multiple ISs contribute to the change in evenness.

A comparison of Pielou's and Simpson's indices was also performed on IS analysis done in WAS patients' PBMCs in a clinical trial long-term follow-up of WAS gene therapy[22] (Figure 5). Even though Pielou's index seems to be more variable than Simpson's, the longitudinal tracking of both indices is consistent with the absence of any notable clonal dominance in this study.

DISCUSSION

The analysis of retroviral or lentiviral vector ISs in gene therapy provides a large amount of complex sequencing and genomic and statistical data that present a challenge for the medical interpretation of gene therapy results. Monitoring the gene-modified cellular diversity in patients is particularly important to follow considering the possible occurrence of clonal dominance with clinical impact. Considering that many different indicators of population diversity have been used in published studies,[16–23] the purpose of the present work was to seek an index, insensitive to the effects of richness and sample size, thus allowing intra- and inter-study comparisons, with meaningful clinical significance. We herein propose to use Pielou's or Simpson's indices to obtain normalized or relative measures of evenness, which is a biologically meaningful aspect of diversity. Here, the analysis of published gene therapy and experimental datasets and *in silico* modeling studies have shown that the popularly used Shannon index is not sufficiently robust as a single indicator, as it varies according to sample richness and size as shown by us and also by others.[24] This Shannon entropy is not useful alone to predict the risk associated with increased clonal dominance in patients with proliferative disorders. While Del Core et al.[24] propose to apply an additional model to reduce the confounding effects on Shannon, we propose to use existing indices. Pielou's and Simpson's indices are robust and measure evenness of clonal abundance in samples. Being scaled between 0 and 1, these indices also provide calibrated values that facilitate the interpretation of results. Based on the reanalysis of gamma-retroviral and lentiviral gene therapy trials in which leukemias occurred or not, we propose that a Pielou's index value of 0.5 may define an alert threshold of low evenness in samples with clinical importance. Simpson's index value could not be modeled from the publication datasets as all information was not available to calculate it, but this index should also provide similar threshold values. The comparison between Pielou's and Simpson's indices shows that Pielou can be more biased by very low sample sizes than Simpson and that Simpson's index is less dynamic and less sensitive to clonal abundance changes than Pielou. Otherwise, the behavior of these two indices is rather comparable.

Other values are important to consider to better interpret results of clonal abundance in gene therapy. The estimation of the number of gene-modified cells (i.e., population richness or population size) is a well-recognized challenge in gene therapy.[32] Counting the numbers of unique ISs is not enough, technical bias exists, and sampling is almost always incomplete. When retrieving ISs after random DNA shearing, it is possible to estimate the size of cellular clones by counting the different linker positions (i.e., different DNA fragments) obtained for each unique IS.[32] Sample richness depends on the sampling effort as the number of expected ISs is proportional to sample size, amount of material, and information obtained (number of cells, DNA quantity, DNA sequencing method, and throughput). As we showed, the observed sample richness may not be a sufficient metric

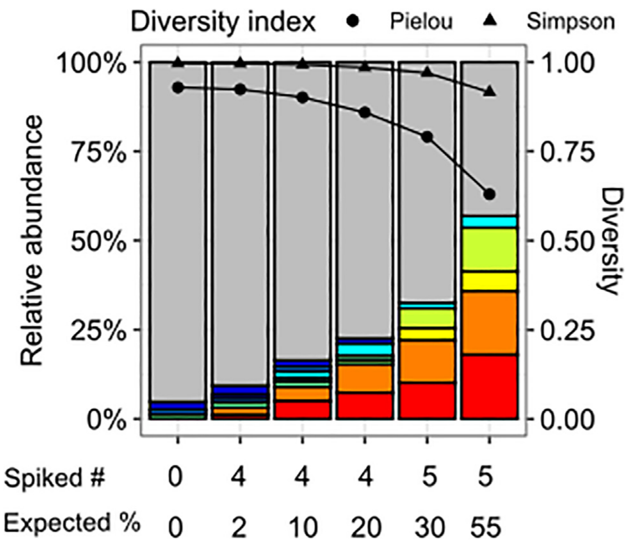

Figure 4. Comparison of Pielou and Simpson indices in polyclonal populations with increasing contribution of a subset of insertion sites

Vector insertion site (IS) clonal contributions is analyzed from a published dataset[27] in which DNA from a polyclonal population of cells transduced with a lentiviral vector (1.5 average vector copy number) is spiked with increasing amounts of DNA from lentiviral standard clones. ISs were obtained by Illumina sequencing following DNA sonication, LM-PCR, and analyzed by the "soniclength" R package (see Corre et al.[27] for details). Under each condition represented in the bar graph, "Spiked #" represents the number of unique ISs added in the population, and "Expected %" represents their cumulated expected abundance. The left axis represents the relative abundance in stacked bars. ISs contributing to ≤1% are in gray, and other colors represent major clones (abundance on left y axis). The right y axis represents the value of diversity indices calculated for each condition.

to estimate the polyclonality over time, across patient or trials if sampling effort varies. Thus, the Chao1 estimator is useful to estimate the size of populations of gene-modified cells.[32] Chao1 provides a higher estimate of population size by using the information on the number of times that ISs are detected or not in each sampling. If all clones are detected several times in a sample, sampling effort may be saturating, indicating that no other clones are expected if sample size increase. Otherwise, other clones are expected from additional or larger samples indicating a large polyclonality or an undersampling issue. Reporting both the observed and the predicted richness using the Chao1 is then of particular interest to interpret clonal abundance in the light of sample completeness.

Diversity indicators are useful in many fields of population studies as reviewed recently with microbiome analyses.[33] The medical importance of normalizing the analyses of diversity in gene-modified cell populations in gene therapy is obvious. It is useful for safety evaluations of integrative vectors or to follow treatment effects as suggested by CAR-T cell studies[12] or to track population dynamics as shown in the case of antiviral immune responses.[34] Normalizing or calibrating the evenness index is essential in gene therapy to express the level of clonal abundance of gene-modified cells in a reproducible and clinically meaningful manner. The use of Pielou's or Simpson's indices and the identification of threshold alert values such as a Pielou <0.5 may facilitate the medical interpretation of gene therapy protocols biosafety. Below this value, diversity should be closely monitored through additional parameters and longitudinal verifications.

MATERIALS AND METHODS

Data and scripts

Data processing and graphical representation were done using R software (4.0.2). Diversity indices were computed using specific libraries (Vegan 2.5.7, ineq 0.2.13). Scripts are available at https://github.com/gcorre/GeneTherapyClonalDiversityIndex_study.

Formulas

Pielou's index named "*J*" in the formula also referred to as the Shannon's equitability index or the Shannon evenness is defined as the ratio of the observed diversity (*H*) by the maximum diversity (*Hmax*) expected considering the number of observed clones (*S*) (i.e., if they were all equi-abundant) with the following formula:

$$J = \frac{\mathrm{H}}{Hmax} = \frac{H}{log_n(S)}$$

$$H = -\sum_{i=1}^{S} p_i \times \log\left(p_i\right)$$

Where, H is the Shannon index, S the number of species, n is the log base, and p_i is the relative abundance of clone i.

The Simpson index was calculated using the following:

$$D = 1 - \sum_{i=1}^{S} p_i \times p_i$$

Gini was determined using Brown's formula:

$$G = \frac{2\sum_{i=1}^{n} i \times y_i}{n \times \sum_{i=1}^{n} y_i} - \frac{n+1}{n}$$

Where n is the number of clones and y_i the abundance of the ith clone ranked in increasing value.

CHAO1

$$\widehat{S} = S + \left(\frac{f_1\left(f_1 - 1\right)}{2 \times f_2}\right)$$

Where, S is the total number of observed clones, and f_1 and f_2 are the number of clones observed once and twice respectively.

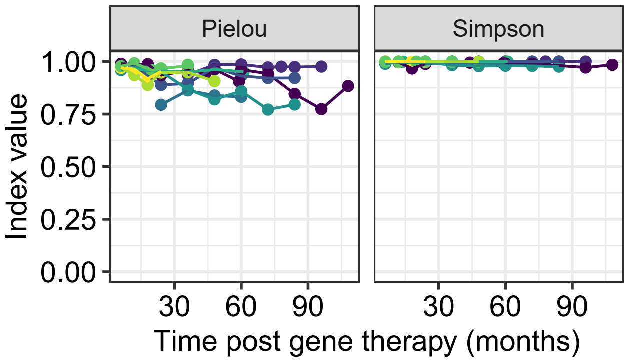

Figure 5. Comparison of Pielou and Simpson diversity indices in a WAS gene therapy study

PBMC samples diversity from the long-term follow-up study of WAS patients[22] were analyzed with Pielou and Simpson indices.

Horvitz-Thompson correction:

$$\widehat{H} = -\frac{\sum_{i=1}^{S} c \times p_i \times \log\left(c \times p_i\right)}{\left(1 - \left(1 - c \times p_i\right)^S\right)}$$

Here, $c = 1 - \left(\frac{f_1}{S}\right)$ and S the total number of observed clones, with f_1 observed once.

In silico simulation of clonal dominance

Clonal dominance was simulated using the R software in order to evaluate different richness and evenness metrics. At T0, we generated N clones, which all have the same relative abundance. Each of them is attributed a similar proliferation rate, except one that is attributed a higher value to mimic a selective advantage and to generate a progressive clonal dominance. At each step of the simulation, the relative abundance of each clone is updated according to the proliferation rate, and the proliferation rate for the next time point is updated with a random value from the normal distribution $\mathcal{N}(0, 1)$. The proliferation rate of the "malignant" clone was chosen so that clonal dominance raised progressively and reached its maximum after 20 pseudotime steps. Random sampling with replacement was performed 20 times for different sample sizes (one-tenth to 10 times the total number of clones). Diversity indices were estimated for each sampling and a summary calculated for graphical representation (mean and standard deviation).

Datasets analyzed

The Shannon diversity index value of different participants to *ex vivo* gene therapy clinical trials listed in Table 1 were extracted from these publications using either the information in the text, tables, or figures. A logarithm base change was applied to Shannon index values when necessary to compare the different datasets.

DATA AVAILABILITY

Data are available upon request. Scripts are available at https://github.com/gcorre/GeneTherapyClonalDiversityIndex_study.

SUPPLEMENTAL INFORMATION

Supplemental information can be found online at https://doi.org/10.1016/j.omtm.2023.05.003.

ACKNOWLEDGMENTS

The authors would like to thank Dr. F. Bushman for critical review of the manuscript and acknowledge the contribution of patients, clinicians, and scientists involved in the studies presented. The authors would like to acknowledge the financial support of AFM/Telethon, the French Muscular Dystrophy Association.

AUTHOR CONTRIBUTIONS

G.C. and A.G. conceived and wrote the study. G.C. performed modeling experiments.

DECLARATION OF INTERESTS

The authors declare no competing interests.

REFERENCES

1. Cavazzana, M., Bushman, F.D., Miccio, A., André-Schmutz, I., and Six, E. (2019). Gene therapy targeting haematopoietic stem cells for inherited diseases: progress and challenges. Nat. Rev. Drug Discov. *18*, 447–462. https://doi.org/10.1038/s41573-019-0020-9.
2. Morris, E.C., and Stauss, H.J. (2016). Optimizing T-cell receptor gene therapy for hematologic malignancies. Blood *127*, 3305–3311. https://doi.org/10.1182/blood-2015-11-629071.
3. Rivière, I., and Sadelain, M. (2017). Chimeric antigen receptors: a cell and gene therapy perspective. Mol. Ther. *25*, 1117–1124. https://doi.org/10.1016/j.ymthe.2017.03.034.
4. Cattoglio, C., Pellin, D., Rizzi, E., Maruggi, G., Corti, G., Miselli, F., Sartori, D., Guffanti, A., Di Serio, C., Ambrosi, A., et al. (2010). High-definition mapping of retroviral integration sites identifies active regulatory elements in human multipotent hematopoietic progenitors. Blood *116*, 5507–5517. https://doi.org/10.1182/blood-2010-05-283523.
5. Wu, C., Li, B., Lu, R., Koelle, S.J., Yang, Y., Jares, A., Krouse, A.E., Metzger, M., Liang, F., Loré, K., et al. (2014). Clonal tracking of rhesus macaque hematopoiesis highlights a distinct lineage origin for natural killer cells. Cell Stem Cell *14*, 486–499. https://doi.org/10.1016/j.stem.2014.01.020.
6. Six, E., Guilloux, A., Denis, A., Lecoules, A., Magnani, A., Vilette, R., Male, F., Cagnard, N., Delville, M., Magrin, E., et al. (2020). Clonal tracking in gene therapy patients reveals a diversity of human hematopoietic differentiation programs. Blood *135*, 1219–1231. https://doi.org/10.1182/blood.2019002350.
7. Biasco, L., Pellin, D., Scala, S., Dionisio, F., Basso-Ricci, L., Leonardelli, L., Scaramuzza, S., Baricordi, C., Ferrua, F., Cicalese, M.P., et al. (2016). In vivo tracking of human hematopoiesis reveals patterns of clonal dynamics during early and steady-state reconstitution phases. Cell Stem Cell *19*, 107–119. https://doi.org/10.1016/j.stem.2016.04.016.
8. Adair, J.E., Enstrom, M.R., Haworth, K.G., Schefter, L.E., Shahbazi, R., Humphrys, D.R., Porter, S., Tam, K., Porteus, M.H., and Kiem, H.P. (2020). DNA barcoding in nonhuman primates reveals important limitations in retrovirus integration site analysis. Mol. Ther. Methods Clin. Dev. *17*, 796–809. https://doi.org/10.1016/j.omtm.2020.03.021.
9. Tucci, F., Galimberti, S., Naldini, L., Valsecchi, M.G., and Aiuti, A. (2022). A systematic review and meta-analysis of gene therapy with hematopoietic stem and

progenitor cells for monogenic disorders. Nat. Commun. *13*, 1315. https://doi.org/10.1038/s41467-022-28762-2.

10. Bushman, F.D. (2020). Retroviral insertional mutagenesis in humans: evidence for four genetic mechanisms promoting expansion of cell clones. Mol. Ther. *28*, 352–356. https://doi.org/10.1016/j.ymthe.2019.12.009.
11. Cavazzana-Calvo, M., Payen, E., Negre, O., Wang, G., Hehir, K., Fusil, F., Down, J., Denaro, M., Brady, T., Westerman, K., et al. (2010). Transfusion independence and HMGA2 activation after gene therapy of human beta-thalassaemia. Nature *467*, 318–322. https://doi.org/10.1038/nature09328.
12. Nobles, C.L., Sherrill-Mix, S., Everett, J.K., Reddy, S., Fraietta, J.A., Porter, D.L., Frey, N., Gill, S.I., Grupp, S.A., Maude, S.L., et al. (2020). CD19-targeting CAR T cell immunotherapy outcomes correlate with genomic modification by vector integration. J. Clin. Invest. *130*, 673–685. https://doi.org/10.1172/JCI130144.
13. Thompson, A.A., Walters, M.C., Kwiatkowski, J., Rasko, J.E.J., Ribeil, J.A., Hongeng, S., Magrin, E., Schiller, G.J., Payen, E., Semeraro, M., et al. (2018). Gene therapy in patients with transfusion-dependent beta-thalassemia. N. Engl. J. Med. *378*, 1479–1493. https://doi.org/10.1056/NEJMoa1705342.
14. Bystrykh, L.V., Verovskaya, E., Zwart, E., Broekhuis, M., and de Haan, G. (2012). Counting stem cells: methodological constraints. Nat. Methods *9*, 567–574. https://doi.org/10.1038/nmeth.2043.
15. Daly, A., Baetens, J., and De Baets, B. (2018). Ecological diversity: measuring the unmeasurable. Mathematics *6*, 119.
16. Boulad, F., Maggio, A., Wang, X., Moi, P., Acuto, S., Kogel, F., Takpradit, C., Prockop, S., Mansilla-Soto, J., Cabriolu, A., et al. (2022). Lentiviral globin gene therapy with reduced-intensity conditioning in adults with beta-thalassemia: a phase 1 trial. Nat. Med. *28*, 63–70. https://doi.org/10.1038/s41591-021-01554-9.
17. Aiuti, A., Biasco, L., Scaramuzza, S., Ferrua, F., Cicalese, M.P., Baricordi, C., Dionisio, F., Calabria, A., Giannelli, S., Castiello, M.C., et al. (2013). Lentiviral hematopoietic stem cell gene therapy in patients with Wiskott-Aldrich syndrome. Science *341*, 1233151. https://doi.org/10.1126/science.1233151.
18. Biffi, A., Montini, E., Lorioli, L., Cesani, M., Fumagalli, F., Plati, T., Baldoli, C., Martino, S., Calabria, A., Canale, S., et al. (2013). Lentiviral hematopoietic stem cell gene therapy benefits metachromatic leukodystrophy. Science *341*, 1233158. https://doi.org/10.1126/science.1233158.
19. Braun, C.J., Boztug, K., Paruzynski, A., Witzel, M., Schwarzer, A., Rothe, M., Modlich, U., Beier, R., Göhring, G., Steinemann, D., et al. (2014). Gene therapy for Wiskott-Aldrich syndrome–long-term efficacy and genotoxicity. Sci. Transl. Med. *6*, 227ra33. https://doi.org/10.1126/scitranslmed.3007280.
20. Hacein-Bey Abina, S., Gaspar, H.B., Blondeau, J., Caccavelli, L., Charrier, S., Buckland, K., Picard, C., Six, E., Himoudi, N., Gilmour, K., et al. (2015). Outcomes following gene therapy in patients with severe Wiskott-Aldrich syndrome. JAMA *313*, 1550–1563. https://doi.org/10.1001/jama.2015.3253.
21. Kohn, D.B., Booth, C., Kang, E.M., Pai, S.Y., Shaw, K.L., Santilli, G., Armant, M., Buckland, K.F., Choi, U., De Ravin, S.S., et al.; Net4CGD consortium (2020). Lentiviral gene therapy for X-linked chronic granulomatous disease. Nat. Med. *26*, 200–206. https://doi.org/10.1038/s41591-019-0735-5.
22. Magnani, A., Semeraro, M., Adam, F., Booth, C., Dupré, L., Morris, E.C., Gabrion, A., Roudaut, C., Borgel, D., Toubert, A., et al. (2022). Long-term safety and efficacy of lentiviral hematopoietic stem/progenitor cell gene therapy for Wiskott-Aldrich syndrome. Nat. Med. *28*, 71–80. https://doi.org/10.1038/s41591-021-01641-x.
23. Wang, G.P., Berry, C.C., Malani, N., Leboulch, P., Fischer, A., Hacein-Bey-Abina, S., Cavazzana-Calvo, M., and Bushman, F.D. (2010). Dynamics of gene-modified progenitor cells analyzed by tracking retroviral integration sites in a human SCID-X1 gene therapy trial. Blood *115*, 4356–4366. https://doi.org/10.1182/blood-2009-12-257352.
24. Del Core, L., Cesana, D., Gallina, P., Secanechia, Y.N.S., Rudilosso, L., Montini, E., Wit, E.C., Calabria, A., and Grzegorczyk, M.A. (2022). Normalization of clonal diversity in gene therapy studies using shape constrained splines. Sci. Rep. *12*, 3836. https://doi.org/10.1038/s41598-022-05837-0.
25. Pielou, D.P., and Pielou, E.C. (1967). The detection of different degrees of coexistence. J. Theor. Biol. *16*, 427–437. https://doi.org/10.1016/0022-5193(67)90065-3.
26. Morris, E.C., Fox, T., Chakraverty, R., Tendeiro, R., Snell, K., Rivat, C., Grace, S., Gilmour, K., Workman, S., Buckland, K., et al. (2017). Gene therapy for Wiskott-Aldrich syndrome in a severely affected adult. Blood *130*, 1327–1335. https://doi.org/10.1182/blood-2017-04-777136.
27. Corre, G., Seye, A., Frin, S., Ferrand, M., Winkler, K., Luc, C., Dorange, F., Rocca, C.J., and Galy, A. (2022). Lentiviral standards to determine the sensitivity of assays that quantify lentiviral vector copy numbers and genomic insertion sites in cells. Gene Ther. *29*, 536–543. https://doi.org/10.1038/s41434-022-00315-8.
28. Wang, Y., Bergelson, S., and Feschenko, M. (2018). Determination of lentiviral infectious titer by a novel droplet digital PCR method. Hum. Gene Ther. Methods *29*, 96–103. https://doi.org/10.1089/hgtb.2017.198.
29. Chao, A. (1987). Estimating the population size for capture-recapture data with unequal catchability. Biometrics *43*, 783–791.
30. Berry, C.C., Nobles, C., Six, E., Wu, Y., Malani, N., Sherman, E., Dryga, A., Everett, J.K., Male, F., Bailey, A., et al. (2017). INSPIIRED: quantification and visualization tools for analyzing integration site distributions. Mol. Ther. Methods Clin. Dev. *4*, 17–26. https://doi.org/10.1016/j.omtm.2016.11.003.
31. Chao, A., and Shen, T.J. (2003). Nonparametric estimation of Shannon's index of diversity when there are unseen species in sample. Environ. Ecol. Stat. *10*, 429–443.
32. Bushman, F.D., Cantu, A., Everett, J., Sabatino, D., and Berry, C. (2021). Challenges in estimating numbers of vectors integrated in gene-modified cells using DNA sequence information. Mol. Ther. *29*, 3328–3331. https://doi.org/10.1016/j.ymthe.2021.10.022.
33. Kim, B.R., Shin, J., Guevarra, R., Lee, J.H., Kim, D.W., Seol, K.H., Lee, J.H., Kim, H.B., and Isaacson, R. (2017). Deciphering diversity indices for a better understanding of microbial communities. J. Microbiol. Biotechnol. *27*, 2089–2093. https://doi.org/10.4014/jmb.1709.09027.
34. Truitt, L.L., Yang, D., Espinoza, D.A., Fan, X., Ram, D.R., Moström, M.J., Tran, D., Sprehe, L.M., Reeves, R.K., Donahue, R.E., et al. (2019). Impact of CMV infection on natural killer cell clonal repertoire in CMV-naive rhesus macaques. Front. Immunol. *10*, 2381. https://doi.org/10.3389/fimmu.2019.02381.

OMTM, Volume *29*

Supplemental information

Evaluation of diversity indices to estimate clonal dominance in gene therapy studies

Guillaume Corre and Anne Galy

Supplemental Materials

Supplemental Figures

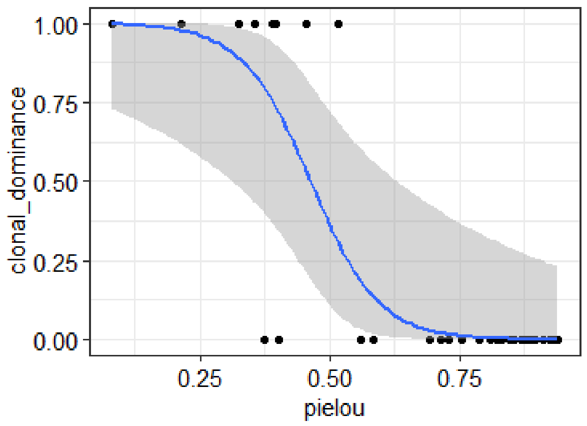

Figure S1 : Regression analysis of Pielou data in Figure 1. A logistic regression curve was generated from the values of the Pielou index from Figure 1 plotted against the clinical status (clonal dominance = 1 in case of leukemia or myelo-dysplastic syndrome, and clonal dominance = 0 ni case of no adverse event). The shaded grey area represents the 95% confidence interval of the regression.

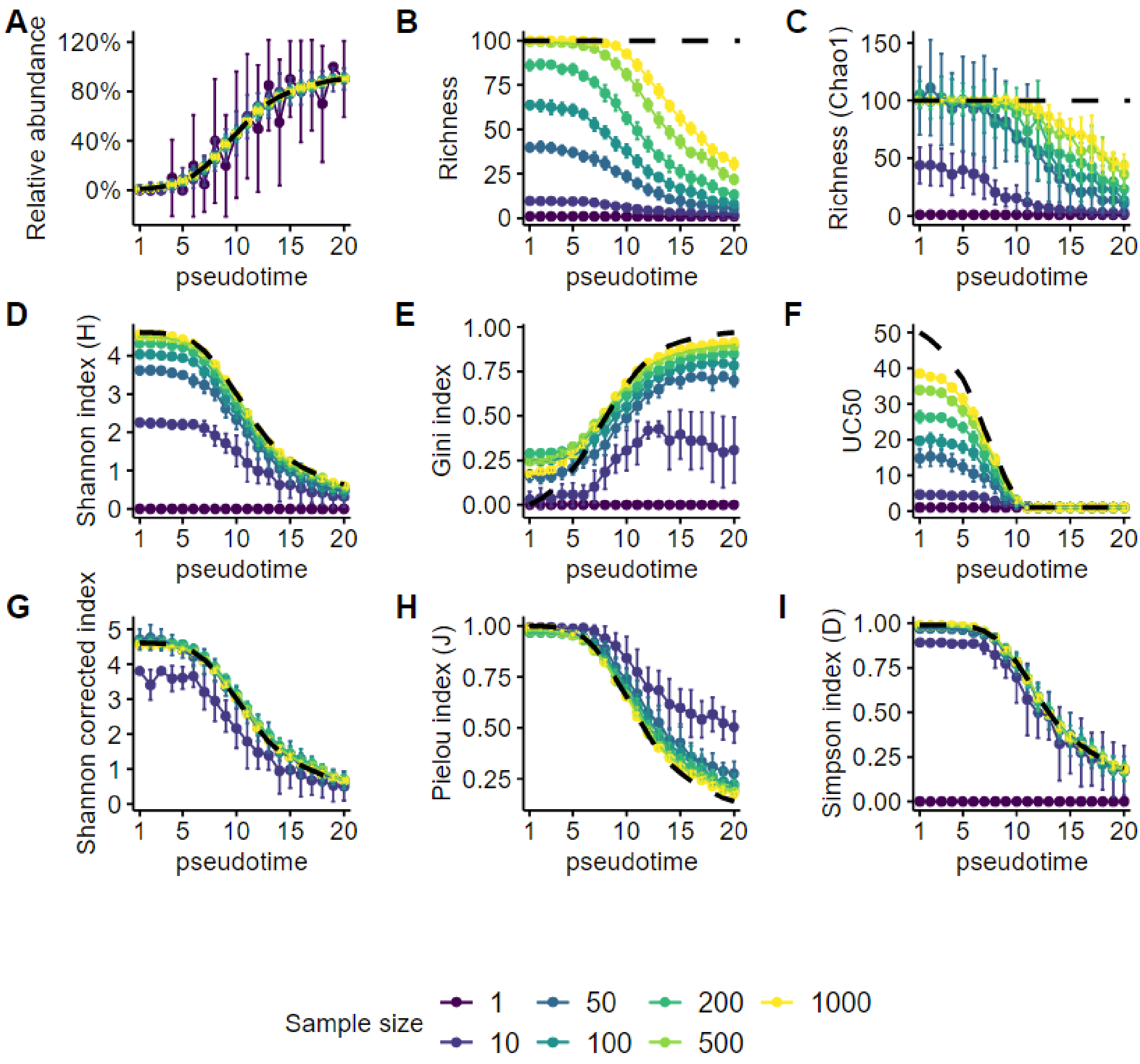

Figure S2: Simulation of a clonal dominance and evaluation of diversity indices with a sample richness of 100 clones. The different parameters are as in Figure 2.

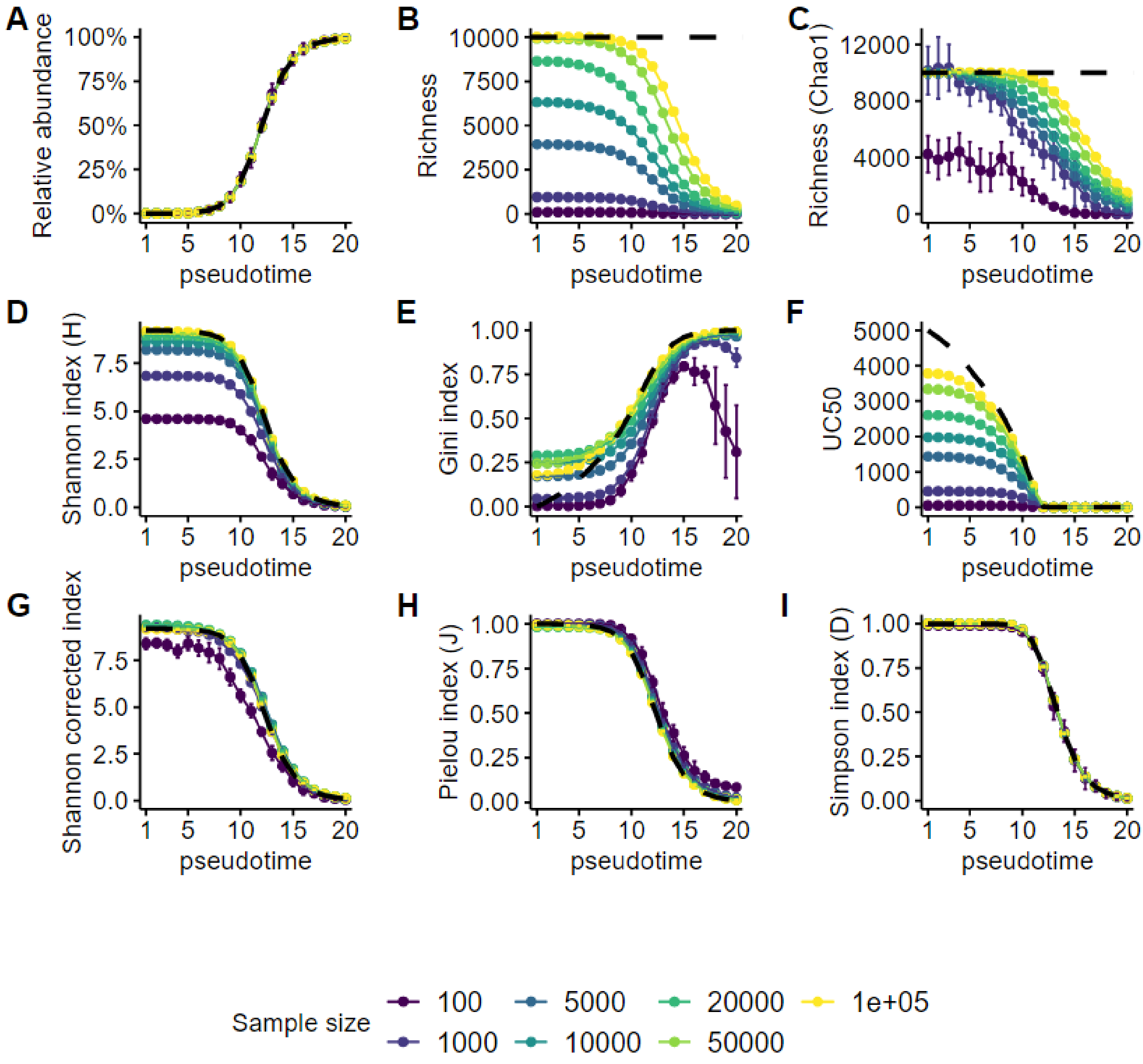

Figure S3: Simulation of a clonal dominance and evaluation of diversity indices with a sample richness of 10,000 clones. The different parameters are as in Figure 2.

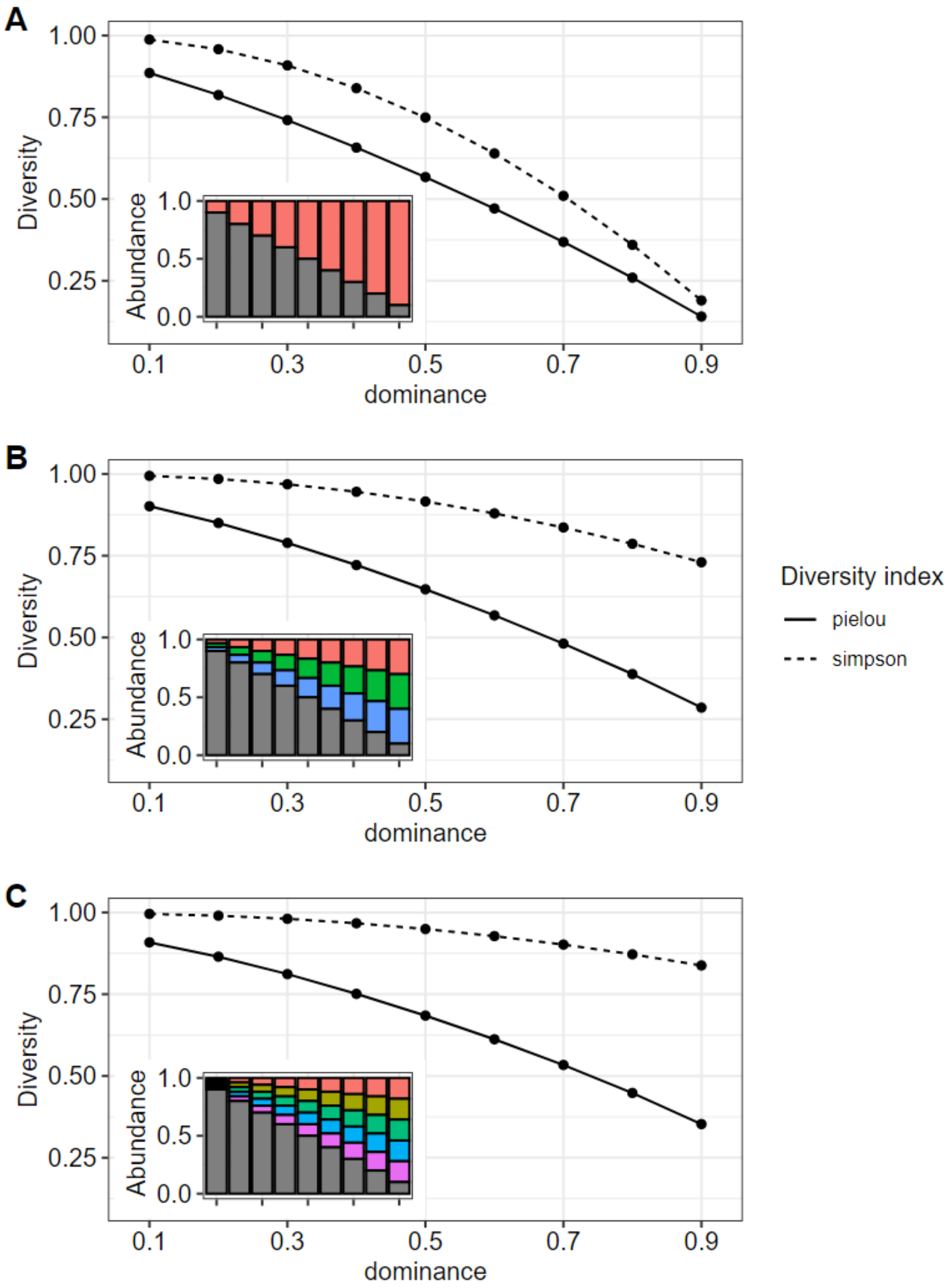

Figure S4 : Comparison of evenness using Pielou and Simpson indices in a population of IS with variable clonal abundance. The IS data set was described in [25]. Panels A-C present the behavior of Pielou and Simpson indices for different numbers of simulated dominant clones: 1 clone (panel A), 3 clones (panel B) and 5 clones (panel C). Inserts present the contribution of dominant clones in color and of the other clones are in grey.
